# Acute Psychosis Consequent to Total Thyroidectomy: An Etiological Conundrum

**DOI:** 10.7759/cureus.9244

**Published:** 2020-07-17

**Authors:** Darwin Kaushal, Nikhil Rajan, Jitender Aneja, Milan Nathvani, Ravindra Shukla

**Affiliations:** 1 Otorhinolaryngology, All India Institute of Medical Sciences, Jodhpur, IND; 2 Otolaryngology - Head and Neck Surgery, All India Institute of Medical Sciences, Jodhpur, IND; 3 Psychiatry, All India Institute of Medical Sciences, Bathinda, IND; 4 Psychiatry, All India Institute of Medical Sciences, Jodhpur, IND; 5 Endocrinology, All India Institute of Medical Sciences, Jodhpur, IND

**Keywords:** thyroidectomy, psychosis, hypothyroidism, hypoparathyroidism, neuropsychiatric

## Abstract

Total thyroidectomy has evolved from a vilipended surgery owing to its high mortality to one with commonly performed surgery with minimal complications. After a total thyroidectomy many patients are left hypothyroid and/or hypoparathyroid, and thus prone to develop neuropsychiatric complications. Although anxiety and depression are the common manifestations, acute psychosis consequent to total thyroidectomy is rarely reported. Herein, we present the case of a 55-year-old female with a massive neck swelling diagnosed as papillary carcinoma of thyroid with bilateral metastatic cervical lymph nodes for which total thyroidectomy with bilateral modified neck dissection was performed. Postoperatively, she developed symptoms of altered sensorium, disorientation, insomnia, agitation, and delusions of persecution as well as suffered from two episodes of generalized seizures. Initially, suspected to be delirium, the persistence of the psychotic symptoms led to revision of diagnosis to psychotic disorder due to another general medical condition.

The acute neuropsychiatric manifestations consequent to major thyroid surgeries may seldom leave the surgeon by surprise. Hence, a multidisciplinary liaising for major thyroid surgeries is the need of hour to avert severe emergencies.

## Introduction

Thyroid surgery has evolved from being banned owing to high mortality to the treatment of choice with less than 1% mortality [1]. Total thyroidectomy is commonly utilized in benign goiter with compression symptoms as well as for thyroid malignancies including papillary carcinomas of size more than 1 cm. Akin to other surgeries, total thyroidectomy has also potential for surgery-related complications and uncommonly neuropsychiatric maladies. Hypothyroidism and hypoparathyroidism consequent to total thyroidectomy may lead to neuropsychiatric symptoms, such as anxiety, depression, cognitive decline, and rarely mania and psychosis. In addition, as a routine clinical practice, thyroid hormone suppression is delayed in view of the need for radioactive iodine (RAI) ablation of the residual thyroid tissue in cases of massive differentiated thyroid cancers [2]. Therefore, this also contributes to the state of hypothyroidism. Despite many advances in the pathophysiological understanding of neuropsychiatric manifestations of hypothyroidism and hypoparathyroidism, there is still a lack of clarity regarding occurrence of psychosis consequent to total thyroidectomy. This may be partly attributed to rare reports of such occurrences. Herein we report an interesting case of acute psychosis and seizures following a total thyroidectomy in a previously euthyroid patient. 

## Case presentation

**Figure 1:**
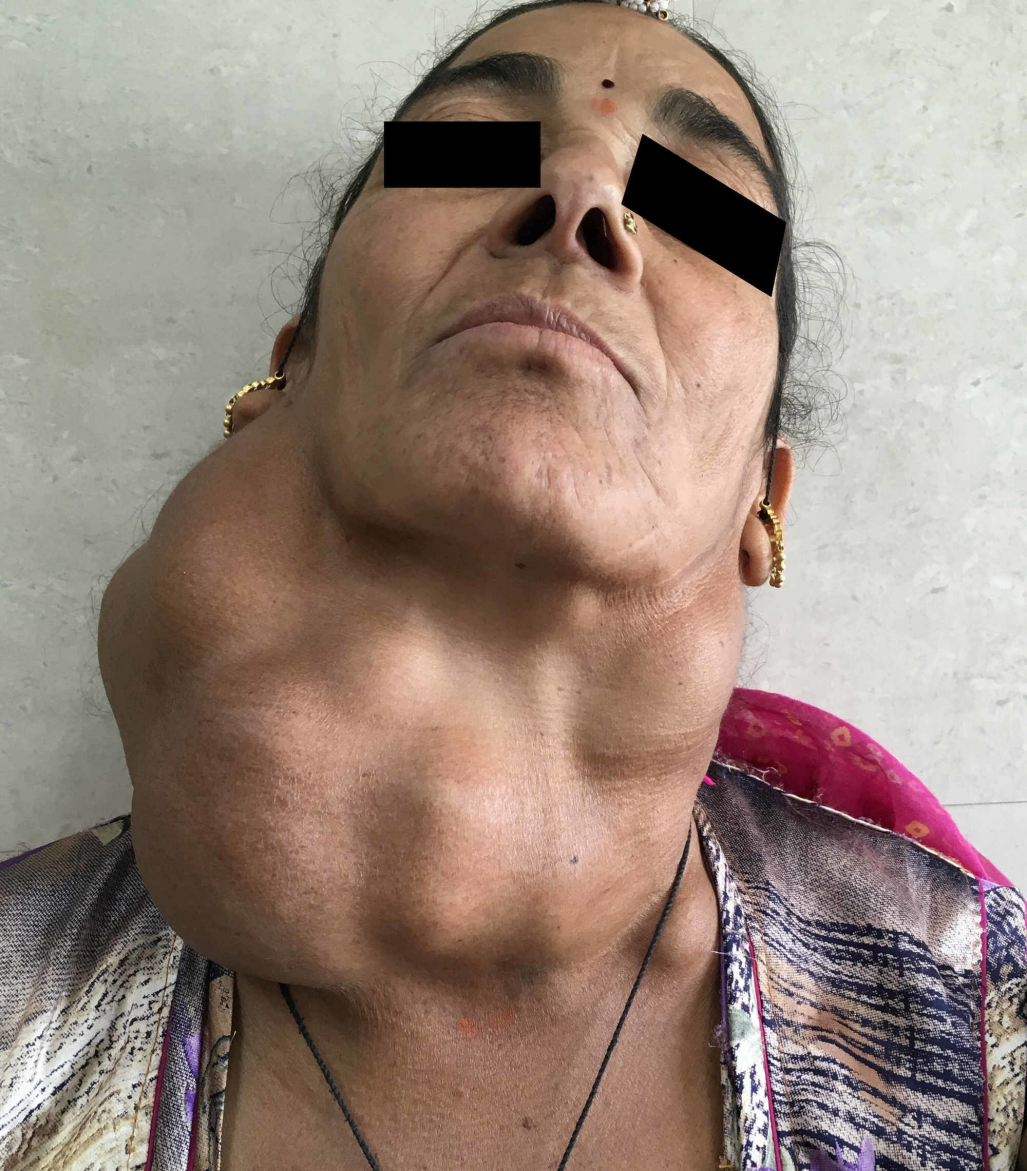
Preoperative picture: Massive lobulated neck swelling measuring 16 x 10 cm extending from sternal notch to mandible, causing deviation of the head to the left.

A 55-year-old lady with gradually progressive mass in neck of 15 years' duration (Figure 1), which was diagnosed as papillary carcinoma of thyroid with metastasis to neck lymph nodes (T4aN1bM0), was admitted for thyroid surgery. She was previously euthyroid and did not report any history of psychiatric illness. The details of preoperative investigations are mentioned in Table 1.

**Table 1:** Results of physical investigation in the preoperative and postoperative period Hb, hemoglobin; WBC, white blood cell count; Neut, neutrophil; Lymph, lymphocyte; Mono, monocyte; Baso, basophil; Eo, eosinophil; MCV, mean corpuscular volume; MCH, mean corpuscular hemoglobin; MCHC, mean corpuscular hemoglobin concentration; AST, aspartate aminotransferase; ALT, alanine aminotransferase; ALP, alkaline phosphatase; BUN, blood urea nitrogen; PT, prothrombin time; CT, control time; INR, international normalized ratio; HbsAg, hepatitis B virus surface antigen; HCV, hepatitis C virus; HIV, human immunodeficiency virus; ELISA, enzyme-linked immunosorbent assay; T3, triiodothyronine; T4, thyroxine; TSH, thyroid-stimulating hormone; PTH, parathyroid hormone; POD, postoperative day.

Name of Investigation	Results
Preoperative	
Complete hemogram	Hb 12.8 g/dL, WBC 4.39 x 10^3^/µL (Neut 56%, Lymph 27.8%, Mono 11.2%, Eo 4.3%, Baso 0.7%), platelet count 239 x 10^3^/µL
Liver function tests	AST 20 U/L, ALT 17 U/L, total bilirubin 0.52 mg/dL, total protein 6.68 g/dL, albumin 3.87 g/dL, globulin 2.8 g/dL, alkaline phosphatase 56 U/L
Kidney function test	BUN 28 mg/dL, creatinine 0.54 mg/dL
Coagulation profile	PT 12.5 s, CT 13.0 s, INR 0.95
Serum electrolytes	Serum sodium 137 mmol/L, potassium 4.06 mmol/L, chloride 105 mmol/L
Chest roentgenogram, postero-anterior view	Normal
HBsAg antigen, HCV antibodies, HIV 1 and 2 ELISA	Non-reactive
Thyroid function test	Free T3 2.18 pg/mL, free T4 0.62 ng/dL, TSH 2.08 mIU/L
Ultrasound neck and thyroid	A very large multiloculated heterogeneous solid cystic lesion seen replacing the both lobes of thyroid. The lesion extended inferiorly up to infra-clavicular location. Echogenic component showed multiple foci of calcification
Ultrasound-guided fine needle aspiration cytology (FNA done from left-sided neck swelling, left lobe of thyroid gland, and right lobe of thyroid gland)	Three FNA samples were withdrawn from different locations of lesion (two from left side and one from the right). Smears from the left side showed features of papillary carcinoma (Bethesda category V) while that from left and right lobes of thyroid showed features of colloid goiter (Bethesda category II)
Serum calcium and phosphorous	10.42 mg/dL (normal range, 8.8-10.6 mg/dL), 4.3 mg/dL (2.5-4.5 mg/dL)
Vitamin D3 levels	38 ng/mL (range, 30-100 ng/mL)
Serum intact parathormone	56 pg/mL (range, 18.5-88.0 pg/mL)
Contrast-enhanced CT of neck and chest	It showed an enlarged thyroid gland that was asymmetrical (right lobe > left lone) and had heterogeneous enhancement with foci of calcifications. Right lobe measured 5.6 x 4.8 x 6.4 cm, and left lobe measured 3.8 x 3.4 x 6.8 cm. Superiorly, the gland extended up to lower border of hyoid, and inferiorly, it reached just above the suprasternal notch. It encased the larynx and trachea, causing deformity. Right internal jugular vein was compressed in the lower part. Also, the left internal jugular vein was compressed by the massive enlargement of gland. Multiple enhancing conglomerated lymph nodes with foci of calcification were noted in bilateral neck that involved all levels and many of them showed cystic changes with enhancing septae. The findings were suggestive of papillary carcinoma of thyroid, with bilateral neck lymph node metastasis.
Postoperative	
Complete hemogram (POD1)	Hb 9.5 g/dL, WBC 8.61 x 10^3^/µL (Neut 85.9%, Lymph 5.6%, Mono 8.4%, Eo 0%), platelet count 138 x 10^3^/µL
Coagulation profile	PT 12.3 s, CT 12.0 s, INR 1.03 (day 1); activated partial thromboplastin time 21.8 s, CT 26.2 s
Peripheral blood film examination (POD1)	Normocytic normochromic profile with few microcytic hypochromic red cells and occasional elliptocytes and spherocytes
Intact PTH	<4.6 pg/mL (POD1)/23.7 pg/mL (POD7)/17.5 pg/mL (POD10) (range, 18.5-88.0 pg/mL)
Serum calcium	9.20 mg/dL (POD1), 8.3 mg/dL (POD3), 7.9 mg/dL (POD7), 8.73 mg/dL (POD10)
Serum phosphorous and magnesium	4.47 (2.5-4.5 mg/dL), 0.8 mmol/L (range, 0.75-1.0 mmol/L)
Serum electrolytes	Sodium 135/138/135 mmol/L (POD1/3/5), potassium 4.02/3.39/3.40 (POD1/3/5) mmol/L, chloride 105/107/104 mmol/L (POD1/3/5)
Thyroid function test	Free T3 <0.30/1.07 pg/mL (POD6 and 30), free T4 0.40/0.96 ng/dL (POD6 and 30), TSH 7.34/41.7 mIU/L (POD6 and 30)
Liver function test	AST 24 U/L, ALT 24 U/L, total bilirubin 0.48 mg/dL, direct bilirubin 0.08 mg/dL, total proteins 5.81 g/dL, albumin 3.20 g/dL, globulin 2.61 g/dL, ALP 64 U/L

**Figure 2:**
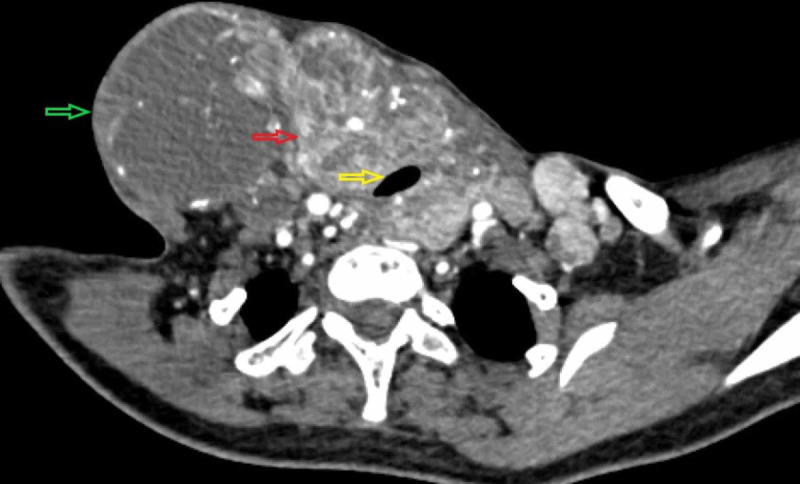
Preoperative axial CT scan section showing massive lymphadenopathy (green arrow) associated with a hypervascular thyroid mass (red arrow) showing microcalcifications, causing narrowing of the trachea (yellow arrow).

Figure 2 shows the preoperative CT image of the neck mass. After obtaining an informed consent, a total thyroidectomy with bilateral modified radical neck dissection type III along with central compartment neck dissection was done, preserving the parathyroid glands. The duration of general anesthesia was 14 hours and approximately 1,200 mL of blood loss occurred during surgery which was managed with transfusion of two units of autologous blood. After surgery, extubation was done uneventfully. On the second day of her ICU stay, the consultation-liaison psychiatry team was called for symptoms of irrelevant talk, agitated behavior, and altered sensorium. On mental state examination, she was conscious, but restlessness and hoarseness of voice were present. The patient, though comprehensible, was disoriented to time, place, and person, and had irrelevant thought content, muttering to self, and intersentence incoherence. Suspicions on the treating team of doing harm to her fulfilled criteria for delusion of persecution. A provisional diagnosis of postoperative delirium was made, and she was prescribed oral haloperidol 0.5 mg twice a day with monitoring of electrocardiogram (ECG). Repeat evaluations for serum electrolytes and blood sugar levels were ordered, which returned normal results. In view of further deterioration and persistence of insomnia, the dose of haloperidol was increased to 2.5 mg twice a day with regular monitoring of her ECG. Following no respite from the symptoms after five days of treatment, haloperidol was substituted with quetiapine 50 mg/d. The dose of quetiapine was built up to 200 mg/d in divided dosages over the next three days and thyroxine supplement 25 mcg/d was started on the seventh postoperative day. On the 10th postoperative day, the patient developed two episodes of generalized tonic-clonic seizures (GTCS). Consultation liaison with neurologist was made, and her detailed neurological examination revealed no neurological deficits and Chvostek and Trousseau signs were negative. There were no signs of meningeal irritation and the fundus examination and contrast-enhanced CT of head were normal. The seizures were initially managed with intravenous midazolam and an infusion of sodium valproate and later on oral phenytoin was started. 

**Figure 3:**
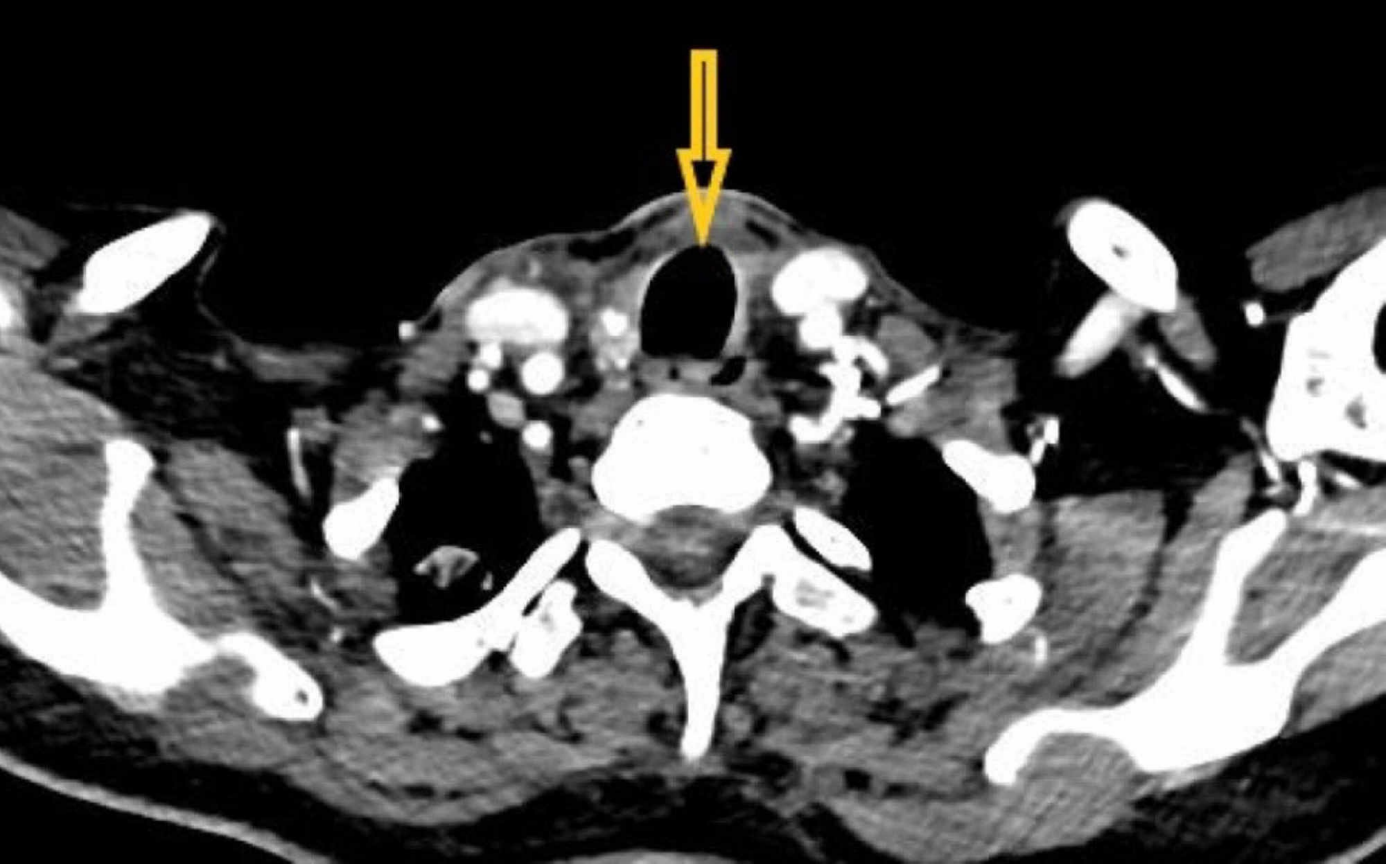
Postoperative axial CT scan section showing clearance of the disease and relief of tracheal compression (shown with the arrow).

In view of remittance of the symptoms of disorientation and persistence of symptoms of delusions of persecution, irritability, agitation, and muttering to self, the diagnosis was revised to psychotic disorder due to another medical condition as per the Diagnostic and Statistical Manual- Fifth Revision (DSM-5) [3]. The dose of quetiapine was further increased to 300 mg/d and the dose of thyroxine was increased to 100 mcg/d on the 15th postoperative day. Her psychotic symptoms remitted in next two months of treatment, and repeat CT scan of neck showed clearance of the disease and relief of tracheal compression (Figure 3).

**Figure 4:**
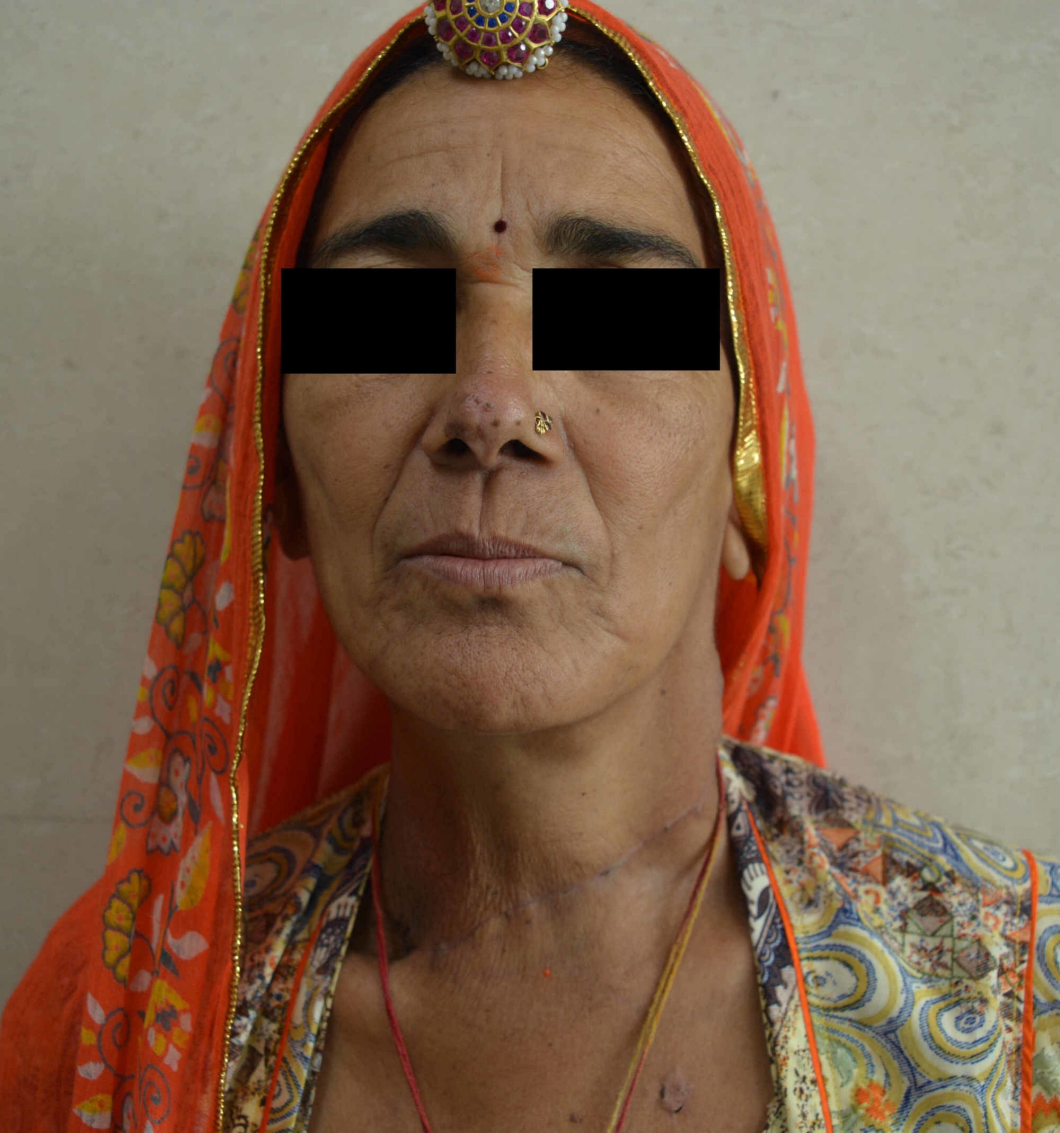
Postoperative picture showing well-healed scar with normalization of the head position.

A whole-body iodine scan done two months after surgery showed two foci of uptake in the neck. Therefore, 100 mCi I131 therapy was administered, following which she is disease-free. At the end of six months, she has achieved her premorbid level of physical appearance (Figure 4) as well as functioning and her antipsychotic medications have been stopped. 

## Discussion

Neuropsychiatric manifestation of primary hypothyroidism is well known, but it often takes weeks to months for the appearance of such symptoms. Furthermore, the acute states of hypothyroidism are commonly associated with anxiety or depression or the worsening of already existing mental illness. But there is minimal literature available for the acute states of hypothyroidism following total thyroidectomy that would have led to psychosis. With best of our efforts, we could find only two case reports of psychosis in patients who underwent thyroidectomy, and only one in which patient developed psychosis in the immediate postoperative period [4,5]. 

Physiologically, triiodothyronine is the active thyroid hormone that is derived from thyroxine by the action of iodinases. Moreover, the central nervous system has the highest T3/T4 ratio which indicates that the brain requires larger amounts of T3 [6]. Therefore, in case of abrupt cessation of thyroid production such as seen after total thyroidectomy and in absence of consequent replacement, which is done in thyroid hormone withdrawal protocols, the brain is likely to experience severe thyroid hormone deficiency. 

Moreover, it has been hypothesized that psychosis occurs due to varied interaction between thyroid hormone and different neurotransmitters such as serotonin, dopamine, and norepinephrine in certain specific brain areas. All these contribute to a diffuse metabolic deficiency in the cerebrum which is also observed in “myxedema madness” [7]. In support to the above are clinical reports of rapid remission of psychotic symptoms with triiodothyronine therapy [7,8]. Unlike the previous case reports, the index patient was euthyroid prior to surgery and the psychiatric symptoms started immediately postsurgery, initially with a picture of delirium that eventually culminated into frank psychosis. In the case reported by Tas et al., the patient was hypothyroid for 15 years and had undergone thyroidectomy for a papillary carcinoma and developed psychosis after three months of surgery [5]. She did not undergo RAI therapy, and her TSH levels were also very high (107.81 mIU/L). However, unlike the index case, she responded quickly to low-dose antipsychotic and thyroid supplement. In addition to the acute onset psychosis, the index patient also had two episodes of generalized seizures which can also be explained by hypothyroidism as reports of seizures in congenital hypothyroidism and myxedema coma exist. Additionally, thyroid abnormalities as an underlying pathology for epilepsy have also been hypothesized [9].

The other possible cause of neuropsychiatric manifestation in this patient could be attributed to the transient hypoparathyroidism which ensued postsurgery. There are high chances of parathyroid getting de-vascularized or damaged unintentionally during a major thyroid surgery involving large neck dissection [10]. Hypoparathyroidism is considered to be transient if the levels of intact parathormone lay low for less than six months following the surgery. It manifests clinically as tingling or numbness, carpopedal spasm, seizures, or neurocognitive dysfunction as well as psychosis. The effects of hypoparathyroidism are mediated by a decline in serum calcium levels, which produce symptoms if they fall less than 7.5 mg/dL or in case of a rapid decline. Serum calcium levels are also influenced by volume overload, malnutrition, and vitamin D levels. In the index case, there was significant decline in intact parathormone levels on the postoperative day 1 along with transient hypoalbuminemia as well as transient hypocalcemia in first 10 days of surgery. However, the parathyroid hormone (PTH) levels and the albumin and serum calcium levels became normal within a week, and the transient nature of these abnormalities might have contributed minimally and thus seem to have contributed minimally in the causation of neuropsychiatric complications in this case. Hence, we propose that the neuropsychiatric manifestations observed in this case could be best explained by an acute onset hypothyroid state after total thyroidectomy. 

## Conclusions

Although anxiety and depression are common consequence of acute states of hypothyroidism, psychosis has been rarely reported. Furthermore, it is a common practice to delay TSH suppression in view of pending RAI ablation in some cases of total thyroidectomy. This in turn may further worsen the state of hypothyroidism which may lead to a range of neuropsychiatric manifestations. Furthermore, the presence of hypoparathyroidism may further confound the clinical picture. Lately, in view of the complications arising due to acute hypothyroidism, it has been suggested that thyroid hormone replacement may be started immediately after total thyroidectomy and recombinant human TSH may be used for RAI scanning which can help in prevention of such complications. Hence, a thyroid surgeon must be watchful of such complication of a major thyroidectomy surgery and a prompt multidisciplinary liaison may help in aversion of emergency. 
